# Spiritual care – ‘A deeper immunity’ – A response to Covid-19 pandemic

**DOI:** 10.4102/phcfm.v12i1.2456

**Published:** 2020-06-15

**Authors:** Nicolette V. Roman, Thuli G. Mthembu, Mujeeb Hoosen

**Affiliations:** 1 Department of Child and Family Studies, Faculty of Community and Health Sciences, University of the Western Cape, Cape Town, South Africa; 2 Department of Occupational Therapy, Faculty of Community and Health Sciences, University of the Western Cape, Cape Town, South Africa; 3 School of Natural Medicine, Faculty of Community and Health Sciences, University of the Western Cape, Cape Town, South Africa

**Keywords:** spiritual care, Covid-19, pandemic, well-being, coping

## Abstract

Coronavirus disease 2019 (COVID-19) has presented unprecedented health challenges across all strata in society throughout the world. The COVID experience has caused us to reflect on quality of life, health and well-being and, just as important, end of life. During this time, spiritual care forms a vital component of holistic health management, especially in terms of coping, coming to terms with illness, suffering and ultimately death. The relationship with the transcendent or sacred has a strong influence on a people’s beliefs, attitudes, emotions and behaviour. Populations, communities, families and individuals have always found solace through their religious or philosophical beliefs during times of personal adversity and widespread anxiety or disaster. Although spiritual care has always been a part of the domain of religious beliefs, a more contemporary perspective is that spiritual care forms part of the human psyche and thus forms part of human care, health and well-being for families, patients and healthcare workers. Spiritual care deals with the provision of compassion and empathy during periods of heightened stress, distress and anxiety within care. This article provides insights into the necessity of providing spiritual care as a means of coping and well-being for families, patients and healthcare workers during the COVID-19 pandemic.

## Introduction

Spirituality is a foundation of all population groups since the beginning of recorded history. It plays an integral component of quality of life, health and well-being both in the general population and those affected by illnesses.^1^ The relationship with the transcendent or sacred has a strong influence on a people’s beliefs, attitudes, emotions and behaviour. Research has shown that families rely on their spirituality for emotional, mental and physical well-being.^2^ Spirituality practices have been recognised as a powerful coping mechanism for dealing with life-changing and traumatic events.^1^ During this global pandemic of Coronavirus disease 2019 (COVID-19), does spiritual care contribute as a coping strategy for practitioners and families?

## The effects of Coronavirus disease 2019 on spiritual care

Coronavirus disease is a serious public health problem that has been confirmed by the World Health Organization (WHO) as a pandemic because of its worldwide spread.^3^ The World Health Organization reports that 4 307 287 people have been tested positive for COVID-19 worldwide in 216 countries.^3^ Out of the confirmed cases, globally there have been 295 101 deaths related to COVID-19.^3^ In the South African context, the Department of Health reports that 403 018 tests were conducted, of which 12 739 people were found to be infected by COVID-19 and 5676 people managed to recover from the disease. The recent COVID-19 crisis has resulted in 238 deaths in South Africa.^4^

The effects of COVID-19 have had a major impact on people’s and front-line health workers’ activities, routines, livelihoods, mental health and well-being.^3,5^ Healthcare workers risk their lives to save people who have COVID-19 while promoting compassionate care. People who have COVID-19 tend to present with severe distress associated with the disease that affects different aspects of their wholeness, including physical, emotional, mental, social and spiritual components.^6^ This means that the healthcare workers need to create a supportive environment that could promote interdependence through a transformative approach of spiritual care.^7^ It further means that all patients and their families should be treated with dignity and be given the voice to express their concerns irrespective of gender, religion, culture, race, sexual orientation and disability.^6,7^

Spiritual care comprises activities that healthcare workers engage in to promote the quality of life and well-being of the clients.^6,8^ The activities that the healthcare workers and people who have COVID-19 engage in include compassionate presence, listening to patients’ fears, hopes and dreams, obtaining a spiritual history, being attentive to all spheres of patients’ lives and their families.^8^ However, in terms of COVID-19, some of the activities, such as the involvement of chaplains and spiritual practices, can be limited because of precautionary measures for infection control.^9^ Spirituality is significant in healthcare because it promotes coping strategies for stress, promotes recovery and resilience and prevents burnout.

## Spirituality for clinical settings

Studies have reported that healthcare practitioners who provide spiritual care to their patients contribute significantly to improve their patients’ overall well-being.^10,11^ Spiritual care is regarded as a life-enhancing factor and a coping resource, which allows patients to deal with adversity in a better way. It may also increase their hopes for the future.^12^ Research reports significantly increased immune functions in response to spiritual care practices.^1^

The current restrictions imposed in South Africa amidst the lockdown prevent free movement and access to normal daily services. South Africans are restricted to the confines of their homes, which may increase levels of anxiety in the general population and even more so in those affected by illnesses. This may negatively affect the health and well-being of many South Africans. These restrictions impede the facilitation of spiritual care in clinical settings.^13^ Spiritual care is based on a bio-psycho-socio-spiritual integrative model that requires a specific set of skills such as active listening, spiritual assessment skills and the ability to refer patients to pastoral care, or other types of intervention services focused on spirituality. For this reason, under the current circumstances, healthcare professionals should be extra-sensitive to the spiritual needs of their patients and their own, as studies have reported that patients’ reliance on spirituality increases during life-changing events.^6,8^ However, this may be problematic as research indicates that whilst many health professionals agree that spiritual care is important for their patients, many are ill-equipped to deal with this aspect.^14^ Collaborative effort may be required to draw on the expertise of those practitioners of spiritual care to support the spiritual care needs of families.^6^

## Spirituality care and the family

The family as the cornerstone of society is a social determinant of health.^15^ For example, when families engage in health-risking behaviour, such as smoking, substance abuse, not exercising or not eating correctly, their behaviours could result in in non-communicable diseases such as diabetes, hypertension, etc. Not only do they place themselves at risk but they also create a negative social atmosphere for their growing children. These patterns could then culminate in health challenges for the next generation. However, families also provide care and support – care of children, the sick and the elderly and support in times of challenges. The most challenging time requiring family support and support to families is during a chronic illness, when one is critically ill and during end of life.^16^

Research has shown that the spiritual care provider plays an important role when families are faced with challenging health risks and the prospect of palliative care.^17^ For example, when families are able to get in touch with a spiritual care provider, they offer a supportive role in the decision-making of a family member who is a patient; they have a sense of peace; they have the opportunity for bereavement and grief counselling and just being able to cope in a very difficult time.^18,19^ The access to spiritual care for the patient and the role of spiritual care for families are clear within the research, but in the case of a pandemic such as COVID-19, where the treatment for the disease becomes limited as more people become sick and the disease is life threatening, there could be challenges to provide this much-needed support. In light of the current global pandemic, examples of the responses of families and communities from countries having faced disasters are discussed.^20^

## Spiritual care in the South African pandemic

The rise in COVID-19 cases in South Africa has necessitated the need for more technologically driven healthcare solutions such as telemedicine. Telemedicine refers to the delivery of healthcare services using information and communication technologies, such as e-mail, telephonic calls, video links and social networks. Globally, many governments and healthcare systems have utilised telemedicine as a primary means of healthcare support during the COVID-19 outbreak. A rise in the number of remotely monitored patients has been reported for most countries.^21^ During this transition from the physical clinical setting to the virtual one, the importance of spiritual care should not be lost or forgotten because it forms part of the holistic approach to deal with the body–mind–spirit aspect of the population.
